# The Development of Emotional and Behavioral Control in Early Childhood: Heterotypic Continuity and Relations to Early School Adjustment

**DOI:** 10.4172/2375-4494.1000204

**Published:** 2015-05-04

**Authors:** Hyein Chang, Daniel S Shaw, JeeWon Cheong

**Affiliations:** 1 Department of Psychology, Sungkyunkwan University, 25-2 Sungkyunkwan-Ro, Jongno-Gu, Seoul, South Korea, Department of Psychology, University of Pittsburgh; Jeewon Cheong, Department of Health; 2 Behavior, University of Alabama, USA; 3 Department of Health Behavior, University of Alabama, USA

**Keywords:** Emotional control, Behavioral control, Heterotypic continuity, Early childhood, School adjustment

## Abstract

We examined heterotypic continuity of emotional and behavioral control (EBC) across early childhood and related early manifestations of EBC to children’s school adjustment in 310 low-income, ethnically diverse boys. Multiple informants and methods were used to measure different indicators of EBC at 18, 24, 42, and 60 months, which were chosen to reflect salient regulatory challenges children face across development. Teachers rated boys’ externalizing and internalizing behaviors, and social skills at 72 months. Results indicated a modest degree of heterotypic continuity of EBC, with different constructs of EBC associated between adjacent time points and, in some instances, across more distant time points. Further, children who had struggled with early EBC demonstrated higher externalizing problems and lower social skills in school. Findings suggest that early deficits in EBC may be a target for early identification and prevention, as they may forecast continued difficulty in later-developing EBC skills and socioemotional problems.

## Introduction

A core developmental task in early childhood is the attainment of self-regulation [1]. Self-regulation is a multifaceted construct that is composed of a set of abilities to monitor and modulate attention, affect, and behavior to meet contextual demands [2]. Research on self-regulation has centered on effortful control and executive function, both of which refer to a higher-order cognitive system that organizes attention and regulates emotional and behavioral impulses [3–5]. Effortful control and executive function have been found to underlie a wide range of child outcomes such as externalizing behavior [6] and school readiness [7]. In addition to effortful control and executive function, a child’s ability to control emotion and behavior has also been operationalized and measured in a variety of ways, including the child’s ability to comply with a request, delay gratification, and engage in harmonious relationships with others [8,9]. Such diversity in methods and contexts that have been utilized to assess the child’s capacities for self-control may support this construct as a critical component of children’s adaptive and maladaptive behavior in many different domains of functioning. For the purposes of this study, we used emotional and behavioral control (EBC) as an umbrella term to encompass diverse behaviors that reflect a child’s ability (or lack of) to regulate impulses in socially adaptive ways. Although there may be varying levels of consensus as to how much each construct represents pure forms of self-regulation, they are all thought to be behavioral manifestations of the child’s ability to control emotion and behavior as described in the subsequent sections.

The predominant scope of prior research on EBC has been elucidating its relation to children’s adjustment. Beginning at the preschool period and extending through early school-age, deficits in EBC have shown to predict socioemotional outcomes [9,10]. Thus tracing the developmental course of EBC may offer useful information for early identification and prevention of an array of problems that are thought to reflect the child’s difficulty with behavioral and emotional control. Although a few recent studies have tracked a single construct of EBC across developmental periods (e.g., inhibitory control) [11], relatively few empirical studies have traced heterotypic manifestations of the child’s ability to control emotion and behavior in early childhood. This is surprising given that many researchers have theorized that the manifestation of EBC may change over time, especially in early childhood when a rapid growth in neurocognitive and motor skills that support self-regulatory processes takes place [12]. As detailed below, temperamental foundation and rudimentary forms of EBC begin to develop in infancy, which become more complex and sophisticated as children mature [1,2] Such transformation of manifestations of a construct (e.g., EBC) across time is referred to as heterotypic continuity [13].

The present study was designed to examine heterotypic continuity in EBC across early childhood and relate early components of EBC to children’s early adjustment at school. We used data from a prospective longitudinal study of low-income boys to identify developmentally salient facets of EBC that have been hypothesized to lead to later difficulties in EBC and related problem behavior. As boys and children of poverty have been found to be at higher risk for deficits in EBC and related problems [14,15] the focus of this study on boys living in poverty provided an opportunity to investigate heterotypic development of EBC and its outcomes in a high risk sample.

### 

#### The development of EBC in early childhood

We first describe EBC behaviors that are salient at different phases in early childhood. It was not our intention to argue that the behaviors described here are the only manifestations of the child’s capacity to control emotion and behavior at those times, but they all reflect critical manifestations of EBC during respective developmental periods. For example, during the toddler period oppositionality and aggression are important indicators of EBC, as low levels of those behaviors reflect a child’s capacity to attain control over his/her impulses, which is limited at this age. With the emerging ability to inhibit immediate responses in the third year [1,12], many children show a normative decline in defiant and aggressive behavior and proceed to face more sophisticated regulatory challenges, including the ability to wait and inhibit impulses during mundane situations. While much research has focused on age-specific forms of EBC, there has been relatively little attention to the continuity and stability underlying different regulatory behavior throughout early childhood (i.e., heterotypic continuity).

##### Infancy and toddlerhood

Infants’ negative emotionality has been highlighted as an antecedent of later developing, more complex forms of EBC [16,17]. Negative emotionality is a core component of the construct of difficult temperament and has been defined as the child’s irritability, unsoothability, and intense negative reactions to stressors [18]. Although negative emotionality has been proposed to be a temperament construct that is distinct from the child’s ability to regulate emotion and behavior [19,20], it has also been viewed as a diathesis or vulnerability factor that predicts increased risk for future problems including deficits in self-regulation [21]. Thus, while acknowledging that negative emotionality may represent a reactive as opposed to regulatory aspect of child behavior, we thought it would be important to consider this construct as an early basis for later developing EBC skills. In this study, negative emotionality was evaluated at the first assessment when children were 18 months of age as it was conceptualized as an early foundation for more complex and voluntary EBC observed at later developmental periods.

Toddlerhood marks an important period in children’s emerging ability to control emotion and behavior [1]. In addition to their newly found mobility, toddlers are consciously aware of their own desires and their ability to act upon and change the environment [12]. As a result, children’s behavior in this period becomes more focused and goal-directed than in infancy. Toddlers also have a better sense of others’ intentions and begin to comply with simple requests that have been viewed as an indicator of early EBC [22]. However, toddlers’ compliance is still governed more by pleasure than reason, and with their limited cognitive skills and understanding of their own abilities, they frequently experience frustration and conflict with others [1], and often resist complying with caregivers’ requests for desired behavior. Moreover, as toddlers have not yet learned to manage their distress effectively, they often resort to aggressive outbursts. Indeed, aggression has shown to peak at age 2 [23]. Thus at 24 months, children’s oppositionality and physical aggression were selected to be assessed as developmentally-salient indicators of EBC.

##### Preschool and kindergarten periods

Around 3 to 4 years of age, children begin to develop what Kopp et al. [1] has termed “true self-regulation.” Preschoolers are more able to control early normative impulsivity, comply with requests, and generate adaptive strategies to better regulate their emotion and behavior [24,25]. This has been attributed to a rapid growth in child inhibitory control, which is a central aspect of self-regulation that refers to a child’s ability to inhibit immediate responses [20]. Inhibitory control is also implicated in models of executive function and temperamental effortful control [3,4]. In this study, child inhibitory control was assessed in a delay of gratification paradigm that requires children to wait for a desired reward and reliably elicits frustration [26].

As children mature, the ability to control emotion and behavior becomes critical for successful interactions with others [12]. By 4–5 years, children understand reciprocity, engage in cooperative play with peers, and regulate their behavior to achieve interpersonal goals [27]. However, the peer context introduces a set of new challenges for children who have limited regulatory capacities (e.g., sharing resources and adult attention). In this study, in addition to examining children’s interpersonal regulation with peers, EBC was also observed during a sibling interaction. As sibling relationships often involve the expression of intense emotions, especially in early childhood, and have been associated with the quality of peer relationship [28], sibling interactions were chosen as apt context in which to observe children’s regulatory skills with similar-aged children.

### The continuity of EBC

#### 

##### Homotypic and heterotypic continuity

Many studies have examined homotypic continuity in EBC, focusing on the temporal stability of a particular form of EBC across different developmental periods. Generally, moderate levels of homotypic continuity have been documented for different constructs of EBC. For example, maternal ratings of child inhibitory control at 2, 3, 4, 5, and 7.5 years have shown moderate cross-time correlations [11]. Similar degrees of continuity have also been reported for parental ratings of negative emotionality and effortful control in early childhood [29]. Using laboratory tasks, Kochanska and colleagues [6,30] have also found moderate levels of continuity in effortful control in toddlerhood and preschool period.

**Figure 1:**
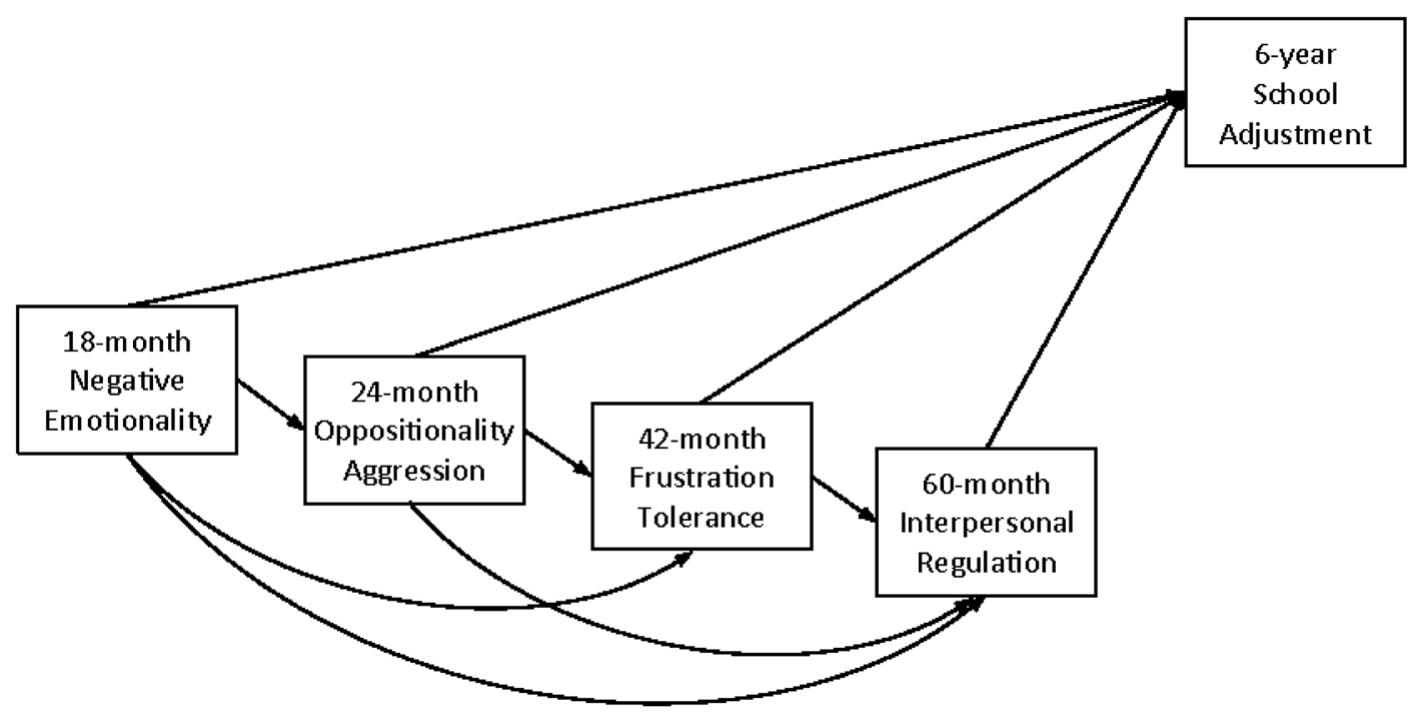
Path model of EBC and school adjustment.

Relatively few studies have investigated heterotypic continuity of EBC with a focus on changing manifestations of EBC over time. Associations between different measures of EBC (i.e., heterotypic continuity) are generally weaker than those between same measures of EBC over time (i.e., homotypic continuity). Previous studies on heterotypic continuity have typically examined relations between children’s early negative emotionality and later forms of EBC. For example, easily frustrated infants (i.e., high negative emotionality) continued to struggle with their ability to control emotion and behavior as toddlers, displaying higher levels of noncompliance compared to their less easily frustrated peers [17]. Additionally, an inverse relationship between negative emotionality in toddlerhood and effortful control in preschool has been documented for both maternal [29] and observational measures [6,30]. However, no study to date has examined heterotypic continuity among developmentally-salient manifestations of EBC across multiple time points in early childhood. Thus, in this study, relations of multiple pairs of distinct EBC behavior were modeled simultaneously in a path model (Figure 1). Associations between both adjacent and distant time points were included with an aim of advancing our knowledge of heterotypic development of EBC in early childhood.

##### Multi-method assessment of EBC

Although it is widely accepted that incorporating information from multiple sources leads to a richer understanding of the phenomenon [31], measures of child behavior from different informants or methods often do not converge highly. In their seminal meta-analysis of cross-informant correlations, Achenbach et al. [32] documented that ratings on an identical measure by informants from the same context (e.g., two parents) are moderately associated, whereas ratings by informants from different settings (e.g., a parent and a teacher) are only modestly related. Other studies have found cross-informant correlations of similar sizes [31]. Some nonsignificant associations have also been reported, as in Kerr et al. [31] study in which child behavior reported by parents was not consistently correlated with ratings by teachers and lab examiners.

Cross-method convergence is even weaker than cross-informant convergence because the constructs are assessed with different methods that also often involve different contexts and informants. Specifically, studies have documented varying degrees of agreement between parent ratings and laboratory observations, two of the most commonly used methods in research with children. For example, modest associations have been found for maternal and observational indicators of negative emotionality [33] and effortful control [14,30] in toddlers and preschoolers. Nonsignificant cross-method convergence have also been reported for children’s negative emotionality [34], effortful control [34], and venting behavior [35], all of which represent dimensions of EBC in this study.

Currently, there is no gold standard about how best to composite discrepant data from multiple sources [36]. In this study, despite our expectations of low and modest correlations between multi-method measures of EBC, we thought it may be important to aggregate different indicators of EBC, as they likely reflected variability in child behavior across contexts and informants [31]. However, in cases when associations were modest across method and/or informant, we also considered examining different methods separately, an issue that is described further in the analysis overview.

##### Cumulative development of EBC

**Figure 2:**
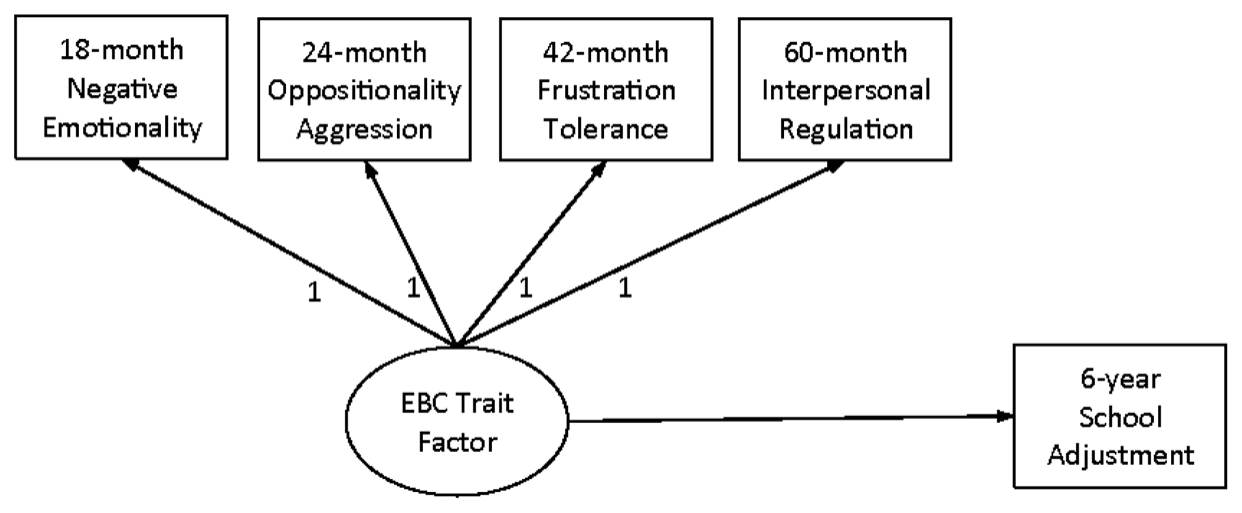
State-Trait (S-T) model of EBC and school adjustment.

The development of an organism is “cumulative,” in that earlier development serves as a foundation for later development [13]. Thus children who experience more difficulty with early EBC are at higher risk for continued struggle with EBC at later years, although the particular regulatory skills that they struggle with may change with development. As described earlier, such heterotypic continuity of EBC was examined in a path model in this study. A related question, however, is whether there is a component of EBC shared among heterotypic indicators of EBC. To address this cumulative aspect of EBC, we explored different analytical strategies such as a state-trait model (Figure 2).

### EBC and school adjustment

School transition is stressful for children. As children enter formal schooling, they are now faced with an increased class size and child-to-teacher ratio, explicit goals for academic progress, and expectations to suppress impulses and get along with others [27]. Children who have had deficits in early EBC may be at a higher risk for problem behavior because dimensions of EBC are so vital to both instrumental and social success in school [9,37]. In this study, we hoped to expand prior findings by examining how heterotypic manifestations of children’s early EBC relate to their school behavior as rated by teachers. Teachers are a valuable source of information as they observe children in situations that often provoke strong reactions and have developed norms about child behavior due to their experience working with many children [38]. Teacher data also represented a new context and informant for assessing child behavior from the methods used to assess earlier EBC.

### The Present Study

Our goals were to examine heterotypic continuity of children’s EBC in early childhood, highlighting different dimensions of EBC that become increasingly salient at different phases of development, and to investigate whether early EBC are related to children’s socioemotional outcomes in the transition to school. We expected that heterotypic continuity of EBC would be evident from the beginning of toddlerhood to the kindergarten period, albeit modest based on the variability of child behavior and the use of multiple informants and methods. It was also hypothesized that children who had deficits in early EBC would be more likely to demonstrate higher levels of externalizing and internalizing behavior, and lower levels of social skills.

Multi-method and multi-informant data were used from a longitudinal study of boys from low-income families. Although the sample was restricted to boys because its original goals focused on overt antisocial behavior, boys have also been shown to experience more deficits in their ability to control emotion and behavior than girls [14,30]. Children of economically disadvantaged families are also known to be exposed to stressors that negatively impact their neurological, cognitive, and affective development [15]. Furthermore, it has been suggested that the effects of child risk factors on future outcomes (e.g., antisocial behavior) may be particularly pronounced for children living in poverty [39]. Therefore, the focus of this study on low-income boys provided an opportunity to investigate heterotypic development of EBC and its outcomes in a sample of children at most risk for deficits in EBC and related problems.

## Method

### Participants and Procedures

Participants were 310 boys who were recruited from the Women, Infant, and Children (WIC) clinics in the metropolitan Pittsburgh area as part of a longitudinal study on child vulnerability and resiliency in low-income families [40]. At initial assessment, the sample was primarily European American (51%) and African American (39%). About one third of the families were single-parent households and two thirds of mothers had education of 12 years or less. Sixty-eight percent of the families were living below the federal poverty line (mean per capita income=$2,892/year).

Families participated in regular laboratory and home visits at 18, 24, 42, and 60 months of age. At each assessment, parents completed a set of questionnaires on their child and family, and engaged with their sons in structured activities that varied in stress level to allow examination of child behavior across contexts (e.g., teaching and clean-up tasks, free play, the Strange Situation). At 60 months, the closest-age sibling of the target child was also invited to participate in an hour-long sibling interaction task. At 6 years, teachers contributed ratings of boys’ problem behavior and social skills. Retention rates were generally high, with 89–96% of the original sample completing assessments at subsequent time points. Families who dropped out from the study did not differ from the rest of the sample in demographic qualities or earlier measures of child behavior. Data for school outcomes were available for 202 children (65% of the sample). Again, children with versus without teacher data did not differ from each other in any study variable. For the purposes of this study, boys who participated in at least two of the assessments were included. The analytical sample (n=298) was not significantly different from the full sample in child or family characteristics.

### Measures

#### 

##### SR in early childhood

At 18, 24, 42, and 60 months, developmentally-salient constructs of EBC were measured by observation and parental report. At each age, a single index of EBC was generated by aggregating observational and questionnaire data.

##### Negative emotionality at 18 months

Child negative emotionality was observed at the 18-month lab visit using one molecular and three global ratings based on approximately 70-minute-long videotapes of mother-son interactions (mean interrater reliability=.87) [41]. For the molecular rating, the percent of time spent fussing and crying was recorded by dividing seconds of fussing and crying by total seconds spent on tasks. Additionally, after watching each entire videotape, coders made global ratings of the amount and intensity of the child’s fussing and crying, and the child’s difficulty compared to others his/her age on 5-point scales (1=low; 5=high). As in Owens et al. [41] study, three scores were standardized and aggregated into a single index of observed negative emotionality (α=.91).

Mothers also provided ratings of their child’s negative emotionality using the 7-item Difficultness factor (e.g., “amount of fussing and crying in general”; α=.81) of the Infant Characteristics Questionnaire [33] and eight items that assess negative emotionality (e.g., “gets angry when doesn’t get his way”; α=.82) of the Toddler Behavior Checklist [30]. To aggregate the two maternal reports, scores from the ICQ and the TBC were standardized and averaged (r=.59, p < .01). The final EBC composite for 18 months was created by standardizing and averaging negative emotionality scores from maternal and observational data (r=.30, p < .01).

##### Oppositionality and aggression at 24 months

Examiners and mothers contributed ratings of toddlers’ oppositional and aggressive behavior. The 24-month assessment began at the family’s home where for approximately one hour parents were interviewed about the child, and the parent-child dyad was observed in less structured tasks than in the lab. After driving families to the lab, mothers and sons completed a similar series of tasks in the lab that had been completed for the 18-month assessment, the entire assessment lasting about 4 hours including transportation time. After the entire assessment, examiners completed a 4-item child global rating scale based on their impression of the child during the visit. Each item was rated on a 4-point scale. For purposes of this study, two items that assess the child’s levels of compliance (1=repeatedly gets in trouble, is disobedient, noncompliant; 4=noticeably cooperative and responsive to directions) and aggression (1=unaggressive; 4=severely aggressive) were used. Two scores were aggregated into a single composite with the compliance item reversed (α=.72).

Maternal perception of the child’s oppositionality and aggression was evaluated using the Child Behavior Checklist for ages 2-3 (CBCL/2-3; Achenbach, 1992) and a few additional items from the TBC. For this study, nine individual items that address the child’s oppositional and aggressive behavior (e.g., “disobedient,” “hits others”) were selected and summed (α=.75). The final EBC composite for 24 months was created by standardizing and averaging the oppositionality and aggression scores from the examiner and maternal reports (r=.11, ns). A low correlation was somewhat expected as studies have documented weak, sometimes nonsignificant, convergence across method and context. To check whether the maternal rating and the examiner rating showed differential associations with other EBC constructs in the model, we estimated the path model (Figure 1) separately by informant (i.e., maternal report only vs. examiner report only). Although the values of coefficients differed to some degree (e.g., the βs from 24-month EBC to 42-month EBC were .12 and .15 in mother-only and examiner-only models, respectively), the results were sufficiently similar to justify their aggregation.

##### Frustration tolerance at 42 months

Children’s ability to tolerate frustration was assessed during a lab visit using a delay of gratification procedure called the cookie task [41]. The boy was placed in a room cleared of toys with his mother who completed a questionnaire. The mother was asked to hold a transparent bag with a cookie inside it in view but out of reach of her child during the task. The child was allowed to have the cookie after three minutes. For every 10-second interval, self-regulatory strategies and child affective displays during the cookie task were rated based on coding systems developed by Grolnick et al. [43], and later adapted by Gilliom et al. [9]. Inter-rater reliabilities based on 30 tapes were: 89–96% agreement for SR strategies (kappas ranged from .64–.79) and 88% agreement for angry affect (kappa was .76). The present study focused on three codes, focus on delay object or task, peak intensity of anger (rated on a 0 to 3 scale), and total time angry (in seconds) because they reflected dysregulated behavior and affect in coping with frustration. The focus on delay object or task code included the child’s behavior that had an intention to end the waiting, such as trying to retrieve the cookie through persuasion, whining, requesting to leave the room, or crying or tantruming. The three codes were standardized and averaged to create a single index of observed frustration tolerance (α=.84).

As for the maternal measure of frustration tolerance, two items from CBCL/2-3 (Achenbach, 1992) were summed: “Wants things now” and “Demands must be met immediately” (α=.70). The final EBC composite at 42 months was created by standardizing and averaging the scores from the cookie task and mother CBCL (r=.22, p < .01).

##### Interpersonal regulation at 60 months

Children’s ability to control their emotion and behavior in interpersonal contexts with similar-age children was assessed based on observations from a sibling interaction task during the home visit and parental reports. The target child and his sibling were videotaped for one hour during which the dyads played with up to three sets of interactive toys (e.g., play set of action figures and materials from the 1995 popular children’s movie, ‘The Lion King’). The dyad was given a choice to switch sets of toys every 20 minutes, but to do so they both had to agree to change before the examiner could introduce the next toy. The task itself was developed by Volling et al. [44], with the current coding system adapted from the authors’ original coding manual [45] for more details about the task, codes, and reliability). For the purpose of this study, global ratings of target negative reactivity, destructive conflict, and intensity of conflict were used. Target negativity reflects how much the target child negatively reacted or overreacted when provoked by the sibling (e.g., a child who constantly whines would get a higher score). Intensity of conflict was defined as the level of negative affect (e.g., yelling, crying, physical aggression) present throughout the interaction. Destructive sibling conflict reflected the extremes of the negative conflict sequences (e.g., presence of many long conflict sequences). The ratings were made on 4-point scales with higher score representing more negative reactivity or sibling conflict. The three codes were aggregated to obtain a composite of observational measure of interpersonal regulation at age 60 months (α=.81). Only the data from children whose sibling was between one year younger and four years older were included in the analysis (n=182) after it was found that the quality of interaction changed when siblings under age 4 or over age 9 were involved. The restriction in age range was chosen so that the siblings would more likely be playmates rather than caretakers, which occurred with much older or much younger siblings. Of this sample, 28 were one year younger, 49 one year older, 48 two years older, 40 three years older, and 17 four years older than the target child. Ninety-seven (54%) of the siblings were boys. Age differences and sex of the sibling were not significantly related to the three codes used in this study (all ps > .05).

Mothers and alternate caregivers (n=177; 80% were biological father of the child) contributed ratings of children’s EBC in interpersonal contexts by completing the Social Skills Rating Scale [46]. Specifically, the Self-Control factor of the SSRS was used, which included items on the child’s ability to regulate themselves in interpersonal situations that may provoke negative reactions (e.g., “Responds appropriately when pushed or hit by other children”; α=.77 and .76 for mother and alternate caregiver reports, respectively). Maternal and alternate caregivers’ scores were averaged to obtain a composite score of parental report of interpersonal regulation (r=.40, p < .01). For children whose alternate caregivers did not participate, only maternal reports were used. The final EBC composite for 60 months was created by standardizing and averaging the scores from the sibling task and parental reports of SSRS Self-Control (r=.28, p < .01).

##### School adjustment

At 6 years, teachers contributed ratings of multiple aspects of boys’ school behavior on the following measures.

##### Externalizing and internalizing behavior

Teachers completed the Teacher Report Form for ages 5-18 (TRF/5-18; Achenbach, 1991) on which they rated their student on a 3-point scale based on his behavior within the past six months. In this study, the broadband Externalizing (α=.96) and Internalizing Problems (α=.87) scales were used.

##### Social skills

The Social Skills Rating Scale [46] is a 30-item measure of children’s ability to conform to social standards in academic and interpersonal contexts. The SSRS is composed of three factors: Cooperation (α=.92), which measures children’s ability to comply to rules (e.g., “Puts work materials or school property away”), Assertion (α=.85), which measures children’s ability to assert their needs in appropriate manner (e.g., “Appropriately questions rules that may be unfair”), and Self-Control (α=.91), which measures children’s ability to control their emotions and behavior in challenging situations (e.g., “Responds appropriately to teasing by peers”). The SSRS factors were analyzed separately to examine the relations of early EBC to different aspects of social skills.

### Analysis overview

Following preliminary analyses (Table 1), substantive research questions were addressed in a structural equation modeling (SEM) framework. In SEM, complex relationships between constructs can be examined simultaneously. For example, dependent variables can also be independent variables within the same model, which was particularly advantageous for investigating heterotypic continuity of EBC in this study because the EBC constructs in the path model were both dependent variables of earlier forms of EBC and independent variables of later forms of EBC (Figure 1). Additionally, SEM allows for the use of latent variables that leads to greater model specificity, such as parceling measurement error from overall model error. Furthermore, many of the current SEM programs employ sophisticated estimation techniques that account for missing data, such as full-information maximum likelihood estimation (FIML), which accommodates missing data by using all available data based on the full sample to estimate each parameter and has been shown to be superior to other missing data methods [47]

An important question in model building was how to composite measures of EBC at each period in a way that best took advantage of the use of a multi-method, multi-informant assessment. Several strategies were considered as there is no gold standard about integrating data from different sources. Initially, we explored the possibility of constructing latent variables of EBC from multiple measures as manifest variables. One problem with this approach was the large discrepancy in factor loadings across observed variables because of low to modest cross-method convergence. For example, for the negative emotionality latent factor, the factor loadings ranged from .76 to .78 for maternal reports but from .27 to .28 for observational measures. In this case, the latent variable would be weighted more based on maternal than observers’ ratings. Although the use of latent variables is generally recognized a strength of SEM, it would have included the disadvantage of disproportionately favoring one method of assessing EBC over another, which was not our goal. In the absence of objective criteria to determine whether one method is more valid or erroneous than another, we decided to aggregate EBC scores across methods, giving them equal weight (i.e., using the mean). As a result, a path model was constructed to examine heterotypic continuity of EBC across adjacent and distant time points, and subsequently, the relations between different forms of EBC and children’s school adjustment (Figure 1). As described above, we also analyzed the model separately by informant at 24 months (mother vs. examiner) when the cross-method association was nonsignificant to examine whether the results were sufficiently similar to justify their aggregation.

Another goal of this study was to examine whether there is a component of EBC shared by different forms of EBC in early childhood using a state-trait (S-T) model. Traditionally, an S-T model is used to differentiate the variability in a psychological construct due to traits from that due to changeable states [48]. By modeling a latent common factor underlying multiple measures of a given psychological construct, the variability accounted for by a trait level factor can be separated from the variability that is more occasion-specific. Those models are similar to intercept-only latent growth models where the average level of a construct across time and individuals’ departure from the average are estimated by the mean and the variance of a latent factor that have factor loadings of 1’s for all indicators. In this study, the trait level or heterotypic continuity of EBC was specified by a common variance shared by all forms of EBC at ages 18, 24, 42, and 60 months (Figure 2). Assuming that the trait level EBC would be related to the age-specific EBC equally, the loadings on the common factor were set to be 1. The residual variance of the EBC measure at each age then represents the variability due to age-specific EBC and other sources such as unmeasured shared psychological characteristics and measurement errors. Thus, it may be difficult to tease apart the variances due to the age-specific EBC and those due to other age-specific sources. However, our focus was on the underlying heterotypic continuity of SR during childhood, rather than age-specific EBC.

Demographic factors such as economic resources and parental education have shown to predict children’s socioemotional outcomes including EBC [15]. Because of observed significant associations with some of the variables analyzed in this study (i.e., 24-month oppositionality and aggression, 60-month interpersonal regulation, and age-6 externalizing and internalizing behaviors; Table 1), family income was included in all models as a covariate.

For all models, Mplus 5.21 with FIML estimation was used [49]. Multiple fit statistics were used to determine how well the specified models approximated the observed covariance structure. Good-fitting models are traditionally indicated by nonsignificant chi-squares. Additionally, Root Mean Square Error of Approximation (RMSEA) values below .05 and the Comparative Fit Index (CFI) value above .90 indicate good model fit.

## Results

### Heterotypic continuity of EBC

#### 

##### Path model

**Figure 3:**
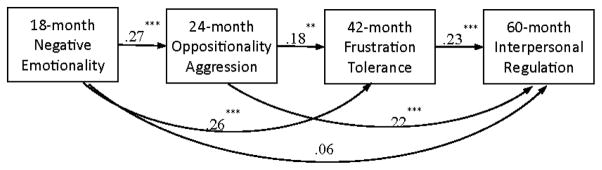
Heterotypic continuity of EBC in early childhood. Note. Standardized coefficients are presented. χ^2^(4)=8.55, CFI=.96, RMSEA=.06. **p < .01, ***p < .001

The path model proposed to examine heterotypic continuity of EBC was first performed with EBC constructs only without school outcomes (Figure 1). The EBC-only model had zero degrees of freedom (i.e., just-identified model) because all possible paths from earlier to later forms of EBC were estimated. Thus fit indices could not be calculated to evaluate the goodness-of-fit of the specified model. The resulting EBC-only model showed good fit to the data, χ^2^ (4)=8.55, p > .05, CFI=.96, RMSEA=.06. As presented in Figure 3, there were modest and significant associations of different forms of EBC between adjacent time points (i.e., 18 months → 24 months, 24 months → 42 months, and 42 months → 60 months) and between distant time points over two time periods (i.e., 18 months → 42 months, and 24 months → 60 months). The path from 18-month negative emotionality to 60-month interpersonal regulation was not significant. The proportions of variance in later EBC explained by the earlier EBC measures were 8%, 13%, and 15% for SR at 24, 42, and 60 months, respectively.

##### Latent S-T model

The S-T model was first estimated with EBC constructs only without school outcomes. This model demonstrated good fit to the data, χ^2^ (8)=5.97, p > .05, CFI=1.00, RMSEA=.00. The mean of the EBC trait factor was zero which was expected as each EBC construct was created by aggregating standardized scores from observational and parental measures. There was significant variability in the trait common factor (σ^2^=.53, p < .001), which indicated that children differed in terms of their overall EBC across early childhood. The proportion of variance in EBC at each age explained by the common trait factor was .30, .27, .24, and .20 for 18, 24, 42, and 60 months, respectively. As a complementary analysis, a common factor model with unconstrained factor loadings was performed. The unstandardized (standardardized) factor loadings of this model were 1.00 (.49), 1.07 (.52), 1.21 (.57), and 0.95 (.51) for 18, 24, 42, and 60 months, respectively (all ps < .001), which were similar to those from the S-T model (all fixed at 1). The Chi-square difference test between the S-T model and the common factor model with unconstrained factor loadings was not significant, χ^2^ (3)=5.97, indicating that constraining all factors loadings to 1 yielded a more parsimonious model.

#### Early EBC predicting school adjustment

##### Path model

Age-6 school outcomes (i.e., externalizing and internalizing behavior, and social skills) were included in the path model shown in Figure 1 to investigate relations between heterotypic continuity of early EBC and children’s school adjustment. Paths from age-specific EBC to school outcomes were estimated to examine whether there were any unique contribution from different components of EBC after accounting for heterotypic continuity in EBC. As shown in Table 2, associations between EBC and school outcomes, again controlling for heterotypic continuity of EBC, showed different patterns depending on the domain of functioning. Specifically, frustration tolerance at 42 months (β=.14, p < .10) and interpersonal regulation at 60 months (β=.12, p < .10) were marginally associated with child externalizing behavior at 6 years. The model explained 9% of the variance in externalizing behavior. Conversely, components of early EBC did not predict children’s internalizing behavior. Early EBC skills were differently associated with different aspects of social skills. Children’s interpersonal regulation at 60 months predicted their levels of cooperation (β=−.15, p < .10) and assertion (β=−.21, p < .01) in school. Of particular interest was the self-control domain as it resembled EBC constructs measured in early childhood. Thus examining relations between early EBC and self-control at early school-age would offer information about whether heterotypic continuity of EBC extends across time and context. Both frustration tolerance at 42 months (β=−.17, p < .05) and interpersonal regulation at 60 months (β=−.24, p < .01) significantly predicted children’s self-control at 6 years. The model accounted for 13% of the variance in children’s self-control.

S-T model. As a final step, relations between the trait level factor in the S-T model and child school outcomes were examined (Figure 2). The results indicated that the trait level EBC that underlies age-specific measures of EBC in early childhood was associated with different domains of school functioning (Table 3). Specifically, the common factor of EBC significantly predicted age-6 externalizing behavior (β=.20, p < .05), explaining 10% of its variance. The global factor of EBC also showed a marginally significant association with cooperation (β=−.18, p < .10). The magnitude of prediction from the global EBC factor was the largest for self-control (β=−.38, p < .001), accounting for 16% of the variance in child self-control. The model did not predict children’s internalizing or assertive behavior in school.

## Discussion

The goals of this study were to examine heterotypic continuity of children’s EBC in early childhood and to relate early forms of EBC to their school adjustment within a sample of low-income boys. As hypothesized, heterotypic continuity of EBC was evident, albeit modest, from the beginning of toddlerhood to kindergarten. Different constructs of EBC (i.e., negative emotionality at 18 months, oppositionality and physical aggression at 24 months, frustration tolerance at 42 months, and interpersonal regulation at 60 months) were significantly associated between adjacent time points and, in some instances, across more distant time points. Further, children who had struggled with early EBC demonstrated more problem behavior in transition to school-age, although specific patterns differed by domains of functioning. Specifically, in both the path model and the S-T model, deficits in early EBC predicted higher levels of externalizing behavior and lower levels of cooperation and self-control. With respect to the child’s ability to be assertive in a socially-acceptable manner, the two models yielded different results. In the path model, EBC in interpersonal contexts at 60 months predicted assertion at 6 years whereas in the S-T model, age-6 assertion was not significantly associated with the common factor that underlie age-specific indicators of EBC in early childhood.

### 

#### Heterotypic continuity of EBC

Heterotypic continuity is a particularly relevant concept in childhood research because rapid developmental changes occur in the early years that may influence manifestations and stability of an underlying construct [13]. This study was an initial attempt to validate the idea of heterotypic continuity in EBC, a notion that has been proposed by many researchers [1,12]. Heterotypic continuity of EBC in early childhood was not expected to be high based on the ongoing development of and changes in neurological and cognitive abilities that support children’s regulatory skills, variability of child behavior across assessment contexts, and the use of multiple informants and methods in this study. The results indicated that different manifestations of EBC, which were chosen for each age to reflect salient regulatory challenges at respective developmental periods, were modestly associated. Our findings are consistent with previous studies that have found modest relations between different forms of EBC in childhood over time [29]. Thus, to some degree, children’s inability to master an earlier regulatory challenge seems to hinder their acquisition of subsequent regulatory skills, reflecting the cumulative nature of development [13].

Moreover, the emerging pattern of relations among heterotypic forms of EBC was more complex than simple continuity from one time point to the next. Earlier levels of EBC were related to later and different EBC skills beyond the adjacent time point. For example, higher levels of negative emotionality at 18 months predicted not only higher oppositional and aggressive behavior six months later, but also lower frustration tolerance two years later even after controlling for the association between earlier forms of EBC. The findings suggest that there may be a unique aspect of negative emotionality that is associated with the child’s ability to endure frustration that is not accounted for by its association with the child’s levels of oppositionality and aggression. Indeed, it has been suggested that the emergence of a child’s ability to inhibit first responses in the third year modulates the stability of earlier, more reactive forms of EBC [20]. An interesting issue for future research will be to explore more specific relations among elements of EBC that contribute to the complexity of overall heterotypic continuity. For instance, can negative emotionality be unpacked into distinct components that have differential relations to oppositionality and aggression versus frustration tolerance?

#### SR and school adjustment

As an empirical test of heterotypic continuity of EBC is a relatively novel addition to the literature, we explored different analytic strategies, including a path model and an S-T model, to capture the heterotypic continuity in EBC. The two models were complementary and estimated different aspects of heterotypic continuity. The path model addressed relations between different pairs of EBC constructs over time, whereas the S-T model more directly examined overall continuity in EBC throughout early childhood. In general, results from the two models were comparable, with the strength of associations between EBC and school-age outcomes being slightly larger in the S-T model. This is not surprising given that the global trait level factor represented a common variance of EBC across all four measurements and thus was a better predictor of children’s later adjustment than EBC specific to certain age.

In both the path model and the S-T model, deficits in early EBC predicted higher levels of externalizing behavior and lower social skills in cooperation and self-control at early school-age. Our findings are consistent with the large body of research supporting inverse relationships between children’s regulatory ability and problem behavior [9,37]. In the path model, it was also possible to determine whether there was any unique contribution from earlier forms of EBC to school-age outcomes other than what was accounted for by heterotypic continuity of EBC. The results indicated that child frustration tolerance at preschool was directly related to teacher reports of externalizing albeit marginally (p < .10) and self-control (p < .01) in the first grade in addition to indirect associations through interpersonal regulation at kindergarten. The findings support the well-established link between effortful control and behavior problems [20]. Additionally, the model for self-control as an outcome was particularly interesting in that this construct was highly similar to earlier measures of EBC at 42 and 60 months (i.e., frustration tolerance and interpersonal regulation). The findings indicated that heterotypic continuity of EBC was evident across time, context (i.e., home to school), and informant (i.e., parents/observers to teachers).

However, early EBC failed to predict child internalizing behavior at school-age. Earlier studies have yielded mixed results regarding the relation between children’s ability to control their emotion and behavior and their internalizing problems. Our findings are in accord with studies that have documented nonsignificant associations between self-regulation and internalizing behavior [50]. However, it may also be possible that EBC measures in this study did not tap areas mores specific to anxiety and depression (i.e., regulation of fear and sadness as opposed to anger).

Interestingly, the path model and the S-T model diverged with respect to child assertion at 6 years as it was significantly associated with interpersonal regulation at 60 months in the path model but not with the common trait factor in the S-T model. We speculated that interpersonal regulation may represent a more complex construct that encompass both EBC and non-EBC components of child behavior in social interactions. Thus the significant relation between interpersonal regulation at 60 months and assertion at 6 years in the path model may be due to factors other than the common factor of EBC that underlie heterotypic measures of EBC. Prior research has also suggested that assertiveness may involve different precursors than other social skills [9]. To test this possibility, the S-T model was reanalyzed with an additional path from 60-month interpersonal regulation to age-6 assertion, which represented the relation between variability in interpersonal regulation not attributable to the trait level EBC and assertion. The results indicated that while the common factor of EBC did not predict assertion (β=.15, ns), the association between residual variance of interpersonal regulation and assertion was significant (β=−. 27, p < .01). These findings provide support for our speculation that the significant path from kindergarteners’ interpersonal regulation to first graders’ assertion in the path model may have been driven by factors other than the child’s ability to control emotion and behavior. The discrepant results also highlight the value of using multiple analytic approaches in examining heterotypic continuity.

#### Contributions, limitations, and future directions

This study has a few limitations. First, the sample was low-income urban boys primarily of European American or African American heritage. Thus our findings may not generalize to girls and children from higher SES families, non-urban environments, or different ethnic groups. With this caveat, our sample allowed a close investigation of EBC among children at high risk for developing deficits in EBC and related problem behavior. Second, although the EBC constructs at each developmental period were chosen to reflect salient regulatory challenges in early childhood, they are not the only forms of EBC observable at those times. Relatedly, if identical, repeated measures of EBC were available at each measurement, we could have performed a more rigorous test of heterotypic continuity of EBC controlling for its homotypic continuity. This would have also allowed us to disentangle reactive versus regulatory aspects of EBC as it has been proposed that constructs in this study may reflect either of those dimensions to different degrees [19,20]. Third, the magnitude of associations between different forms of EBC was modest but consistent with prior research [29]. The fact that our findings are comparable to the few existing studies on heterotypic development of EBC is noteworthy given that we used multiple methods and informants resulting in modest degrees of convergence between observational and parental measures of EBC. Nevertheless, we thought it was important to aggregate different sources of information as they may reflect variability in child behavior across contexts [31,35]. Fourth, because the focus of this study was to elucidate the nature of heterotypic continuity in early EBC and its relations to school outcomes, we did not explore other factors that may be involved in those processes. For example, different forms of EBC may be influenced by and interact with parenting [10,51]. Thus a question for future research will be to investigate the role of parenting and other contextual factors in the heterotypic development of EBC. This may also help explain the modest levels of heterotypic continuity in EBC found in this study and contribute to discovering effective strategies for promoting EBC in young children.

Despite the limitations, the current investigation extends research on the development of early EBC in several ways. Most importantly, this study is among the first to examine heterotypic continuity of EBC beginning as early as the toddlerhood and spanning to early school-age. Following proposed theories on heterotypic development of EBC [1,12], the EBC constructs were selected in light of salient regulatory challenges that children face in the first few years of life. Other strengths of this study include the use of a prospective longitudinal design and multiple methods and informants, and the exploration of different analytical strategies to investigate heterotypic continuity of EBC. Our findings suggest that early deficits in EBC may be a promising target for early identification and prevention as they may be precursors of continued difficulty in later-developing regulatory capacities and related socioemotional behavior.

## Figures and Tables

**Table 1:** Descriptive statistics and bivariate correlations

	N	M	SD	2	3	4	5	6	7	8	9	10	11
Demographic variable													
1. Maternal education	298	12.57	1.51	.28**	−.05	−.06	−.02	−.08	−.00	−.02	.00	.03	.03
2. Family income (per month)	298	1047.79	638.73	--	−.08	−.13*	−.07	−.18**	−.22**	−.15*	.02	−.04	.12
EBC in early childhood													
3. Negative emotionality	298	−.00	.72		--	.27**	.32**	.18**	−.01	.04	.05	.09	−.05
4. Oppositionality/aggression	292	−.01	.76			--	.25**	.29**	.10	.09	−.10	−.02	−.13
5. Frustration tolerance	282	.02	.82				--	.30**	.16*	.09	−.10	−.03	−.23*
6. Interpersonal regulation	272	−.03	.84					--	.18*	.09	−.17*	−.19**	−.29**
School outcome													
7. Externalizing behavior	202	9.41	11.99						--	.47**	−.56**	−.27**	−.68**
8. Internalizing behavior	202	5.87	6.68							--	−.41**	−.48**	−.42**
9. Social skills: cooperation	194	13.13	4.89								--	.53**	.60**
10. Social skills: assertion	195	10.57	4.12									--	.54**
11. Social skills: self-control	195	13.15	4.57										--

* p < .05,

** p < .01

**Table 2:** Path model of early EBC predicting school adjustment.

EBC variable	School outcome									
	Externalizing		Internalizing		Cooperation		Assertion		Self-Control	
	β	R^2^	β	R^2^	β	R^2^	β	R^2^	β	R^2^
Negative emotionality (18mo)	−0.07	0.09	0.00	0.03	0.11	0.05	0.12	0.05	0.04	0.13
Oppositionality and aggression (24mo)	0.04		0.05		−0.08		0.00		−0.05	
Frustration tolerance (42mo)	0.13†		0.06		−0.07		0.00		−0.17*	
Interpersonal regulation (60mo)	0.12†		0.04		−0.15†		−0.21**		−0.24**	

Note. As shown in the path diagram (Figure 1), heterotypic relations between EBC variables were also estimated but not included in the table (see Figure 3). Standardized coefficients are presented. All χ^2^ (4) ≤ 8.72, all CFI ≥ .96, all RMSEA ≤ .06.

† p < .10,

* p < .05,

** p < .01

**Table 3:** State-Trait (S-T) model of EBC predicting school adjustment.

EBC variable	School outcome									
	Externalizing		Internalizing		Cooperation		Assertion		Self-Control	
	β	R^2^	β	R^2^	β	R^2^	β	R^2^	β	R^2^
Intercept	0.20*	0.10	0.14	0.04	−0.18†	0.03	−0.10	0.01	−0.38***	0.16

Note. Standardized coefficients are presented. All χ^2^ (14) ≤ 17.57, all CFI ≥ .97, all RMSEA ≤ .03.

† p < .10,

* p < .05,

*** p < .001
